# Marketing methods for electronic resources in medical libraries: a study on the application of the analytical hierarchy process

**DOI:** 10.5195/jmla.2022.1351

**Published:** 2022-07-01

**Authors:** Rogheyeh Eskrootchi, Mohammad Ali Boroumand

**Affiliations:** 1 eskrootchi.r@iums.ac.ir, Department of Medical Library and Information Science, School of health management and information sciences, Iran University of Medical Sciences, Tehran, Iran; Department of Management Information system, College of Business Administration, California State University San Marcos, CA, USA.; 2 boroumand.m@iums.ac.ir, Department of Medical Library and Information Science, School of health management and information sciences, Iran University of Medical Sciences, Tehran, Iran.

**Keywords:** Databases as topic, information services, libraries, medical, library materials, library services, marketing, marketing of health services, AHP

## Abstract

**Objective:**

Paired with the high cost of providing access to electronic resources in medical libraries, the inefficient use of these resources highlights the need for more efforts to promote these resources than ever before. In this study, electronic resource marketing methods were prioritized and the best strategies were determined using the analytical hierarchy process (AHP).

**Methods:**

Using an analytical survey of officials of medical libraries, the most common methods for marketing electronic resources in libraries were determined and divided into categories of strategies. Five important criteria for marketing strategies were also selected. Using the analytical hierarchy process, pairwise comparisons were performed between the alternatives (i.e., strategies), which were evaluated against the selected criteria. Data analysis was performed using Expert Choice 11 software.

**Results:**

A total of 44 electronic resource marketing methods were identified and categorized into 4 strategies. On average, 43.9% of these methods were used by the surveyed libraries. The analytical hierarchy process showed that simplicity was the most important criterion and that communication networks were the best electronic resource marketing strategy. Home/off-campus access, group training, library search stations, and marketing by individual librarians were the most preferred methods of marketing electronic resources.

**Conclusion:**

With the availability of a variety of different methods for marketing electronic resources, medical libraries must select strategies based on important criteria depending on the characteristics of the library, librarians, and users. Thus, the analytical hierarchy process can be an effective and practical solution to decision-making by mathematically prioritizing the selection of the best strategies from a set of alternatives based on differentially weighted criteria.

## INTRODUCTION

Providing access to valid scientific information is considered one of the primary missions of academic libraries. Due to technological advances, library users often demand services utilizing computer facilities and communication networks [1,2]. In particular, one of medical libraries' primary resources that consumes a large portion of their budget is electronic resources [3]. These resources can be updated quickly and provide users with various extra features regardless of space or time restrictions. Although providing electronic resources facilitates users' access to the latest scientific information, the purchase and provision of electronic resources alone cannot increase visits and resource use [4,5]. Therefore, promoting, monitoring, and controlling usage is not less important than providing access. Due to the enormous annual costs incurred by academic libraries in providing access to electronic resources, it is essential to ensure their effective use [6,7]. Adequate knowledge, enough time, easy access, information literacy training, information acquisition skills, and effective marketing methods also affect the use of electronic resources [6–11]. Using information-based marketing methods, such as providing information to users about electronic resources, can increase their usage [12,13]. Despite some planning done by libraries to promote electronic resources, the impact of some programs is short-lived and cannot alone increase the long-term use of these resources [14]. Therefore, with a coherent long-term plan for marketing electronic resources, an increase in their use could be expected [5,15–17]. To increase the use of electronic resources, libraries must evaluate electronic resource usage regularly, and electronic resource marketing should also receive special attention [18,19]. Currently, there is a growing need to employ new methods of electronic resource marketing in academic libraries in response to internal and external needs for information. The most common methods include social media, email, questionnaires, surveys, websites, and group training [20].

Marketing is an organizational function and a set of processes for creating, communicating, and delivering value to customers and managing customer relationships in ways that benefit the organization and its stakeholders [21]. In some cases, students and even faculty members and university researchers are not sufficiently informed to select the right path in searching for information from various available databases [8,10,11]. Therefore, implementing and strengthening library resource marketing programs could increase the number of visits and use of electronic resources, resulting in the achievement of new successes in providing offline and remote services to users (7,19,22,23). Successful marketing requires knowing enough about users' needs. This can be implemented through creative actions, collaboration of librarians, and the use of various means of communication [24]. However, due to various limitations, it is impossible for libraries to use all available marketing methods, such that each library must choose to focus on certain methods.

The literature indicates that academic libraries use various methods for introducing databases to their users [4,8,17,20,22,25], and some previous studies divided methods of marketing electronic resources into several general categories [26]. Some technological methods of marketing electronic resources, like web advertisement, are widely used by libraries [6]. By contrast, costly methods such as printing and publishing advertising materials or offering incentives such as gift cards or giveaways are less commonly used [4,17]. Also, some methods such as interpersonal communication or the advice of influential people have been positively used in academic settings. In addition, librarians' interactions with users can increase the use of electronic resources [7]. Therefore, it is necessary to use multiple marketing strategies to promote users' access to, and use of, electronic resources.

Libraries vary in terms of the methods used to market electronic resources, which can be due to differences in librarians' views, situations, and various factors [20]. Although a large part of the budget of libraries in Iranian universities of medical sciences is spent on providing access to electronic resources, these libraries may sometimes not pay enough attention to the marketing of these resources [19] or follow a specific marketing strategy [27]. Deciding on the best ways to market libraries' electronic resources is a difficult task given that different circumstances, conditions, or statuses of each library can influence that decision. Therefore, using the analytical hierarchy process (AHP) to mathematically prioritize and select the best strategies from a set of alternatives based on differentially weighted criteria may be an effective and practical solution, as the human brain can only compare a limited number of factors in decision-making [28,29].

In this study, we first identified different electronic resources marketing methods used by libraries in Iranian universities of medical sciences. Next, by examining the opinions of library officials using AHP, we operationally prioritized these methods based on the perceived importance of practical criteria to select the best strategies for marketing electronic resources [30,31].

## METHODS

This study employed an analytical research survey. First, based on the literature, 56 methods of electronic resources marketing were identified [4,7,15,17,22,25,32–34]. In reviewing these methods, duplicate items were removed or integrated into other methods, resulting in the inclusion of 44 methods in a checklist. This checklist was sent to 50 central library officials of Iranian universities of medical sciences, who were asked to indicate whether they used each method and their preference for each method on a scale of 1 to 9. A total of 45 responses were received. The 44 marketing methods were divided into 4 main strategies: training users, physical media, communication networks, and personal interactions [26]. Next, based on the literature, 15 criteria that impact marketing of electronic resources were identified, and 5 essential and practical criteria were chosen according to researcher consensus: time-saving, cost-saving, simplicity, equipment-free, and location-independence.

**Figure 1:**
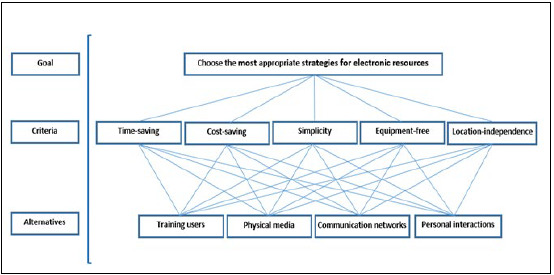
Hierarchical structure for choosing the best strategies for electronic resources marketing.

In this manner, we created an AHP with a hierarchical structure consisting of three levels (Figure 1). The highest level was the final goal or problem that needed to be solved, which was identifying the best strategy for marketing electronic resources. The lowest level consisted of all possible solutions, called alternatives, which were the four main marketing strategies (training users, physical media, communication networks, and personal interactions). The middle level consisted of the criteria by which the alternatives were judged (time-saving, cost-saving, simplicity, equipment-free, and location-independence).

Twenty of the 50 library officials were randomly selected from the previous population and asked to perform pairwise comparisons of the importance of each criterion based on a 9-point scale. A score of 1 indicated equal importance of two criteria, whereas a score of 9 indicated the absolute importance of one criterion over the other [30]. For example, library officials were asked: “what is the relative importance of the cost-saving of implementing a strategy for marketing electronic resources as opposed to its time-saving”? If one criterion X is absolutely more important than another criterion Y and is rated at 9, then Y must be absolutely less important than X and is valued at 1/9 in a pairwise comparison matrix. These pairwise comparisons were carried out for all criteria. Next, using Expert Choice 11 software, the results of pairwise comparisons were used to calculate weights for each criterion based on its relative importance using the relative value vector (RVV). We then formed an option performance matrix (OPM) by calculating the pairwise comparisons for the alternatives. As we had 4 alternatives and 5 criteria, we needed 5 sets of pairwise comparisons to evaluate the performance of each alternative in terms of the 5 criteria. Next, we used standard matrix calculations to produce an overall vector to determine the best strategy by multiplying the RVV by the OPM. Finally, we calculated a consistency ratio (CR) to assess the consistency of participant judgments relative to random judgments. CR values > 0.1 indicate randomness in participants' judgments, which would render the AHP valueless.

## RESULTS

A checklist of 44 electronic resource marketing methods was distributed among library officials, who were asked to indicate whether they used each method and their preference for each method on a scale of 1 to 9. Seven marketing methods were used by more than 80% of libraries, and 16 were used by more than 50% of libraries (Table 1).

**Table 1:** Frequency of use of electronic resource marketing methods.

	Marketing Method	Frequency of Use	Percent of Use
**1**	User guides	44	97.8
**2**	Group training (e.g., workshops)	43	95.6
**3**	Webpage/website	40	88.9
**4**	Banners/posters	39	86.7
**5**	Individual training	38	84.4
**6**	Marketing by individual librarians	38	84.4
**7**	Home/off-campus access	37	82.2
**8**	Online training materials/tutorials/demos	32	71.1
**9**	One-on-one informal appointments	32	71.1
**10**	Library search stations	32	71.1
**11**	Library noticeboard	32	71.1
**12**	Surveys	31	68.9
**13**	Sending emails	28	62.2
**14**	Current user relationship management	26	57.8
**15**	Flyers/brochures	25	55.6
**16**	Phone calls	24	53.3
**17**	Social media	22	48.9
**18**	Administrative letters to individuals	21	46.7
**19**	Lecture on new services	19	42.2
**20**	User feedback forms	19	42.2
**21**	Training by vendors	18	40.0
**22**	Publication of annual training calendar	18	40.0
**23**	Announcement of collection policy	17	37.8
**24**	Invite experts for training	16	35.6
**25**	Marketing by faculty/professionals	16	35.6
**26**	Library newsletter	16	35.6
**27**	Webpage email alerts	15	33.3
**28**	Materials from publisher	14	31.1
**29**	Newsgroups/forums	14	31.1
**30**	Postcards	13	28.9
**31**	Excerpts from news about electronic resources	12	26.7
**32**	Resource of the month	10	22.2
**33**	Collaboration with external institutions	8	17.8
**34**	Bookmarks	8	17.8
**35**	Introduction through the library OPAC	8	17.8
**36**	Computer screen savers	8	17.8
**37**	Network marketing through scientific groups	6	13.3
**38**	Introduction and use campaigns	6	13.3
**39**	RSS feeds	6	13.3
**40**	Giveaways (e.g., pens, notepads, mouse pads)	4	8.9
**41**	Incentives (e.g., gift cards)	4	8.9
**42**	Weblog	4	8.9
**43**	Podcasts	2	4.4
**44**	Wiki	2	4.4
**45**	Other	2	4.4
	**Total**	**89**	

After obtaining the frequency of use for all 44 methods, these methods were categorized into 4 alternatives as potential strategies, and average preference scores were calculated (Table 2).

**Table 2:** Average preference scores of electronic resource marketing methods.

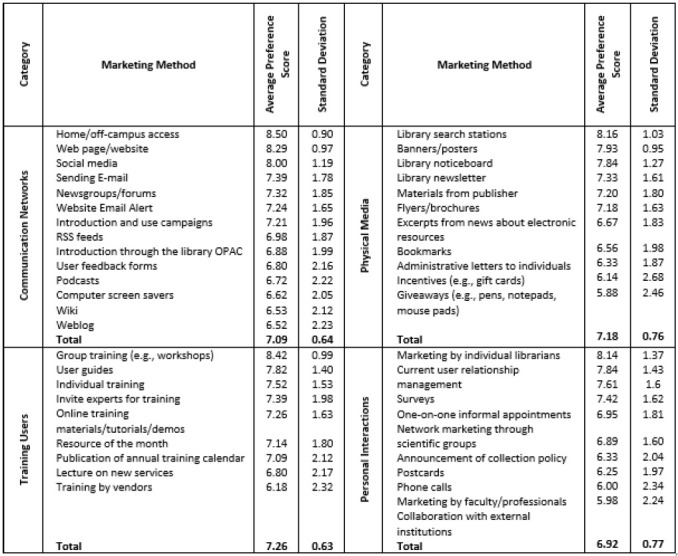

Next, the final weights of each criterion and alternative, which reflect their perceived importance by participants, were calculated using the RVV and OPM, respectively, with Expert Choice software. Table 3 shows a summary of the final scores from this analysis along with their synthesis with respect to the goal, which was calculated as Σ_(k=1)^n▒ 〖x^k a^k, where “x” is the weight of each alternative, “a” is the weight of each criterion, and “n” is the number of criteria in each row. The calculated CR for these data was 0.02, which demonstrates consistency in the participants' judgments relative to random judgments.

**Table 3:** Matrix table comparing criteria versus alternatives.

**Criteria**	**Name**	**Time-saving**	**Cost-saving**	**Simplicit y**	**Equipment-free**	**Location-Independence**	**Synthesis with Respect to Goal**
	Final weight	0.229	0.124	0.323	0.124	0.200	
**Alternatives**	Communication networks	0.391	0.299	0.346	0.173	0.507	**0.361**
	Training users	0.383	0.233	0.319	0.290	0.119	**0.279**
	Physical media	0.142	0.181	0.206	0.278	0.156	**0.187**
	Personal interactions	0.084	0.288	0.129	0.260	0.219	**0.173**
**Total**		1	1	1	1	1	Overall Inconsistency = 0.02

According to library officials, the best strategy for marketing electronic resources is using communication networks, followed by training users, physical media, and personal interactions. After ranking the strategies for electronic resources marketing, the individual methods within each category/strategy were prioritized using the preference scores obtained from library officials (Table 2). The most preferred methods within the communication networks strategy were home/off-campus access, web page/website, and social media. Within the training users' strategy, the most preferred methods were group training, preparation of user guides, and individual training. Within the physical media strategy, library search stations, banners/posters, and noticeboards were the most preferred methods. Within the personal interactions strategy, marketing by individual librarians, current user relationship management, and performing surveys were the most preferred methods.

## SENSITIVITY ANALYSIS

**Figure 2:**
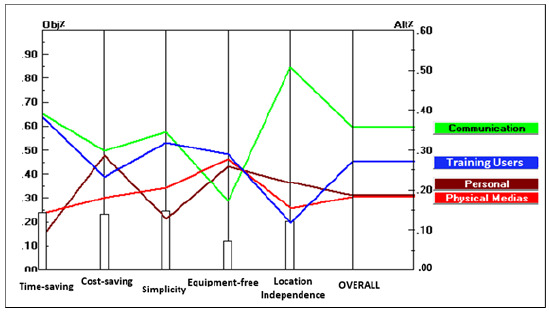
Performance sensitivity graph of alternatives and criteria.

Data sensitivity analysis was performed using Expert Choice software. Figure 2 shows the ranking of each alternative relative to the others based on the perceived importance of each criterion. This performance sensitivity graph shows that with respect to the perceived importance of criteria, communication networks is the best strategy.

Due to the dynamic nature of the sensitivity analysis, any changes in the importance of the criteria would lead to changes in the selected strategies. Thus, using Expert Choice software, we modified the importance of each criterion to determine how the prioritization of strategies changed in response. Increasing the importance of time-saving or simplicity criteria made training users the best strategy. Increasing the importance of the cost-saving criterion made personal interactions the best strategy. Increasing the importance of the equipment-free criterion made physical media and personal interactions the most appropriate strategies. Finally, increasing the importance of location-independence made communication networks the best strategy.

## DISCUSSION

The purpose of this study was to determine the best strategies for marketing electronic resources considering the views of the officials of medical libraries. Other research has also been performed in this area [6,25,35].

Promoting electronic resources is a challenge for librarians [15,36] but can be accomplished using marketing techniques [7,8,10,22]. In our study, not all marketing methods were used in all surveyed libraries due to a lack of knowledge and implementation planning of these techniques. Among the most widely used methods, traditional methods such as user guides, posters, or educational classes were widely used by libraries. In addition, easily accessible methods such as library web pages, free online training materials, email, or library search stations were also widely used. Consistently, previous studies report that most libraries used their websites to advertise electronic resources [6] and that peer education, brochures, and email were widely used [35]. However, we found methods that have a financial burden for libraries, such as printing and publishing advertising materials and incentives such as gift cards or giveaways, are less used, which is also consistent with previous research [4,17].

In addition to the diversity of these methods, libraries should have a specific program to promote electronic resources to increase their use [36]. However, libraries must often prioritize multiple alternatives based on a set of different criteria and select the best options from all available options. Using the AHP, it is possible to prioritize similar alternatives in other areas of academic librarianship, such as resource selection, publishers, library evaluation, educational planning, and outsourcing. When libraries prioritize available alternatives based on various criteria, a hierarchical analysis approach can facilitate comprehensive decision-making.

Our examination of the criteria affecting electronic resource marketing showed that simplicity and time-saving were the most valued criteria; therefore, communication networks was prioritized above training users, physical media, and personal interactions, respectively. However, each method has benefits and limitations. Advantages of communication networks are saving time, saving cost, being simple, and being independent of location, but its weaknesses include a need for equipment. Training users has the advantage of requiring minimal facilities, whereas it is limited by a need for a physical presence and is location-dependent. The primary constraint of using physical media is that their creation is time-consuming, but users do not need special equipment to use them. Also, cost-saving is a more critical aspect of using personal interaction as a strategy, but it requires more time to be implemented. Our results also illustrate that not all methods within each strategy are ranked equally. Some methods such as home/off-campus access, group training, library search stations, websites, social media, and marketing by individual librarians are more preferable, similar to previous findings [20]. By contrast, methods such as using giveaways and collaboration with an external institution are less preferable. Personal interactions receive a higher priority when cost-saving is more important. If libraries have limited availability of computer hardware and software, it may be better to use physical media and personal interaction strategies for marketing. Also, if a library has location constraints, it may better to use a communication networks strategy. Medical librarians should also become more familiar with the principles and skills of information marketing and, consequently, become more motivated and skilled in this area. In addition, as users' information needs change and advances in technology occur, the marketing methods should be synchronized accordingly and changed over time. Appropriate marketing methods are not necessarily costly and can be implemented efficiently using low-cost techniques that rely on communication facilities and the Internet. At the same time, maintaining more traditional methods that help faster face-to-face communication with users should also be considered.

## Data Availability

Data associated with this article are available in the online appendix.

## SUPPLEMENTAL FILES

**Appendix A:** ER Marketing AHP Data Set

**Appendix B:** Research Materials
