# Supplementary material for: Marketing methods for electronic resources in medical libraries: a study on the application of the analytical hierarchy process
Source: J Med Libr Assoc. 2022 Jul 1;110(3):332–9. doi: 10.5195/jmla.2022.1351 (PMC9782590; doi:10.5195/jmla.2022.1351)
Supplement: Supplementary file 2 — Appendix B: Research Materials [file jmla-110-3-332-s02.pdf]

Appendix B: Research Materials

Questionnaire A:

Dear Head of the Library

The questionnaire in front of you is related to the research project entitled "Prioritization of effective methods in marketing electronic resources of libraries of Iranian universities of medical sciences using a hierarchical analysis approach (AHP) which has been developed with the aim of identifying the most effective methods of introducing and marketing electronic resources and has been provided to you as the highest group of scientific resource service providers due to the importance of the opinions of the librarians of these universities. Many thanks and gratitude in advance for taking a few minutes of your valuable opportunity in this way.

Main Categories	Electronic-Resources Marketing Methods	Level of Importance									Current Situation	
		1	2	3	4	5	6	7	8	9	Used	Not Used
Communication-networks	Sending emails											
	Introduction and use campaigns											
	Home/off-campus access											
	Introduction through the library OPAC											
	Newsgroups/ forums											
	Social media											
	Computer screen savers											
	Podcasts											
	RSS feeds											
	User Feedback forms											
	Web page email alert											
	Web page\web site											
	Weblog											
	Wiki											
Personal Interactions	Announcement of collection policy											
	Collaboration with external institutions											
	Marketing by faculty / professionals											
	Marketing by individual librarians											
	Network marketing through scientific groups											
	One-on-one informal appointments											
	Surveys											
	Phone calls											
	Postcards											
	Current user relationship management											
Physical-medias	Banners/ posters											
	Bookmarks											
	Flyers/brochures											
	Giveaways (e.g., pens, notepads, mouse pads)											
	Incentives (e.g., gift cards)											
	Administrative letters to individuals											
	Library noticeboard											
	Library newsletter											

	Library search stations											
	Materials from publisher											
	Excerpts from news about electronic resources											
**Training Users**	Online training materials/ tutorials /demos											
	Group-training (e.g., Workshop)											
	Individual training											
	Invite experts for training											
	Lecture on new services											
	Publication of annual training calendar											
	Resource of the month											
	User guides											

Appendix B: Research Materials

Questionnaire B:

Dear Head of the Library

The questionnaire in front of you is related to the research project entitled "Prioritization of effective methods in marketing electronic resources of libraries of Iranian universities of medical sciences using a hierarchical analysis approach (AHP)" which has been developed with the aim of identifying the most effective methods of introducing and marketing electronic resources. This questionnaire is designed based on a pairwise comparison of electronic resource marketing methods. Many thanks and gratitude in advance for taking a few minutes of your valuable opportunity in this way.

Questionnaire completion guide:

The methods that can be used to market electronic resources are identified based on the literature review and are classified into four general groups as described in the table below:

Category	Communication-networks	Training Users	Physical-medias	Personal Interactions
Marketing Methods	▪Home/off-campus access<br>▪Web page\web site<br>▪Social media<br>▪Sending emails<br>▪Newsgroups/forums<br>▪Web page email alert<br>▪Introduction and use campaigns<br>▪RSS feeds<br>▪Introduction through the library OPAC<br>▪User Feedback forms<br>▪Podcasts<br>▪Computer screen savers<br>▪Wiki<br>▪Weblog	▪Group-training (e.g., Workshops)<br>▪User guides<br>▪Individual training<br>▪Invite experts for training<br>▪Online training materials/ tutorials /demos<br>▪Resource of the month<br>▪Publication of annual training calendar<br>▪Lecture on new services<br>▪Training by Vendors	▪Library search stations<br>▪Banners/ Posters<br>▪Library noticeboard<br>▪Library Newsletter<br>▪Materials from publisher<br>▪Flyers/brochures<br>▪Excerpts from news about electronic resources<br>▪Bookmarks<br>▪Administrative letters to Individuals<br>▪Incentives (e.g., gift cards)<br>▪Giveaways (e.g., pens, notepads, mouse pads)	▪Marketing by individual librarians<br>▪Current user relationship management<br>▪Surveys<br>▪One-on-one informal appointments<br>▪Network marketing through scientific groups<br>▪Announcement of collection policy<br>▪Postcards<br>▪Phone calls<br>▪Marketing by Faculty/ professionals<br>▪Collaboration with external institutions

In addition to the above methods, by reviewing the texts, the main Criteria affecting the use of electronic resources, in the form of 5 main criteria including "Time-saving", "Cost-savings", "Simplicity", "Equipment-free" and "Location-Independence" have been identified and the mentioned marketing methods will be compared in pairs based on these criteria. In each section, please compare the general marketing methods according to the mentioned criteria and determine their importance in comparison with each other.

Appendix B: Research Materials

Pair comparison of general marketing methods of electronic collections in the library

1-Prioritize each of the general marketing methods of electronic resources in terms of "Time-saving" criteria compared to other methods.

	9	8	7	6	5	4	3	2	1	2	3	4	5	6	7	8	9	
Training Users																		Physical Medias
Training Users																		Communication networks
Training Users																		Personal Interactions
Physical Medias																		Communication networks
Physical Medias																		Personal Interactions
Communication networks																		Personal Interactions

2-Prioritize each of the general marketing methods of electronic resources in terms of "Cost-savings" criteria compared to other methods.

	9	8	7	6	5	4	3	2	1	2	3	4	5	6	7	8	9	
Training Users																		Physical Medias
Training Users																		Communication networks
Training Users																		Personal Interactions
Physical Medias																		Communication networks
Physical Medias																		Personal Interactions
Communication networks																		Personal Interactions

3-Prioritize each of the general marketing methods of electronic resources in terms of "Simplicity" criteria compared to other methods.

	9	8	7	6	5	4	3	2	1	2	3	4	5	6	7	8	9	
Training Users																		Physical Medias
Training Users																		Communication networks
Training Users																		Personal Interactions
Physical Medias																		Communication networks
Physical Medias																		Personal Interactions
Communication networks																		Personal Interactions

4-Prioritize each of the general marketing methods of electronic resources in terms of "Equipment-free" criteria compared to other methods.

	9	8	7	6	5	4	3	2	1	2	3	4	5	6	7	8	9	
Training Users																		Physical Medias
Training Users																		Communication networks
Training Users																		Personal Interactions
Physical Medias																		Communication networks
Physical Medias																		Personal Interactions
Communication networks																		Personal Interactions

5-Prioritize each of the general marketing methods of electronic resources in terms of "Location-Independence" criteria compared to other methods.

	9	8	7	6	5	4	3	2	1	2	3	4	5	6	7	8	9	
Training Users																		Physical Medias
Training Users																		Communication networks
Training Users																		Personal Interactions
Physical Medias																		Communication networks
Physical Medias																		Personal Interactions
Communication networks																		Personal Interactions

6- In general, in your opinion, how important are the following Criteria in persuading users and their use of electronic collections in comparison with each other?

**Criteria**	9	8	7	6	5	4	3	2	1	2	3	4	5	6	7	8	9	**Criteria**
Time-saving																		Simplicity
Time-saving																		Equipment-free
Time-saving																		Cost-saving
Time-saving																		location-Independence
Simplicity																		Equipment-free
Simplicity																		Cost-savings
Simplicity																		location-Independence
Equipment-free																		Cost-savings
Equipment-free																		location-Independence
Cost-savings																		location-Independence
